# The effect of health literacy on health investment intention: a cross-sectional study among petrochemical employees in China

**DOI:** 10.3389/fpubh.2024.1358269

**Published:** 2024-06-21

**Authors:** Delong Wang, Ying Wang, Huifen Ma, Shichao Zhao

**Affiliations:** ^1^School of Administration, Shandong Normal University, Jinan, China; ^2^School of Management, Shandong University of Traditional Chinese Medicine, Jinan, China; ^3^School of Medical Management, Shandong First Medical University, Jinan, China

**Keywords:** health literacy, health investment intention, petrochemical industry, health education, occupational health

## Abstract

**Backgrounds:**

In the petrochemical industry, employees are exposed to various health hazards, which pose serious challenges to their health and hinder the sustainable development of the petrochemical industry. Investing in health has proved a potential strategy to enhance general health. However, global health investment is notably insufficient, mainly due to the public’s limited intention to invest in their health. While past research has identified various determinants of health investment intentions, the relationship between health literacy and health investment intention remains somewhat controversial and needs more empirical validation.

**Objectives:**

This study aims to assess the level of health literacy and health investment intention among employees in one of China’s largest petrochemical companies and to explore the effect of health literacy on health investment intention.

**Methods:**

A cross-sectional study was conducted in a petrochemical company. The valid sample size for this study was 39,911 respondents. Data were collected using a designed questionnaire, including socio-demographic information, questions about health investment intention, and the “2020 National Health Literacy Monitoring Questionnaire.” Several statistical analysis methods were employed, including descriptive analysis, Chi-square test, logistic regression, and multiple linear regression.

**Results:**

The study disclosed an average health literacy score of 56.11 (SD = 10.34) among employees, with 52.1% surpassing the qualification threshold. The “Chronic Disease” dimension exhibited the lowest qualification rate at 33.0%. Furthermore, 71.5% of the employees expressed an intention to invest in health, yet a significant portion (34.5%) opted for the minimal investment choice, less than 2,000 RMB. Logistic regression analysis indicated a positive correlation between health literacy and health investment intention (OR = 1.474; *p* < 0.001). This association’s robustness was further indicated by multiple linear regression analyses (*β* = 0.086, *p*<0.001).

**Conclusion:**

The employees’ health literacy significantly exceeds the national average for Chinese citizens, yet the qualified rate in the “Chronic Disease” dimension remains notably low. A majority of employees have the intention to invest in health, albeit modestly. Furthermore, while health literacy does positively influence health investment intention, this effect is somewhat limited. Accordingly, personalized Health education should be prioritized, with a focus on improving chronic disease knowledge and facilitating the internalization of health knowledge into health beliefs.

## 1 Introduction

Occupational Health has become a crucial determinant of the development of the world economy and society, particularly in developing countries (1, 2). Worldwide, an estimated 1.53 million deaths and 76.1 million DALYs were attributable to the included occupational risk factors, accounting for 2.8% of deaths and 3.2% of DALYs from all causes (3). Specifically, in the petrochemical industry, employees are exposed to various health hazards, leading to significant health concerns (4, 5). These issues hinder the sustainable development of the petrochemical industry and, more importantly, pose serious challenges to the health and well-being of employees. Therefore, it is imperative to prioritize the health of petrochemical employees.

Health investment, encompassing preventive healthcare, nutrition, physical activity, etc. (6), presents a viable approach to enhancing overall health. Research substantiates that health investments effectively reduce disease risk, total medical expenditures, and health insurance costs (7). For example, Levi et al. (8) found that future healthcare expenses could be reduced by 6.2 dollars for every dollar invested in preventative healthcare. However, despite its recognized importance, the inadequacy of health investment is quite common. As an illustration, personal and household expenditures on preventive healthcare services constitute approximately 0.33% of the overall GDP and even less than 0.10% in developing countries (9).

The insufficient investment in health can primarily be attributed to the public’s limited intention to invest in their health (10). The health investment intention is influenced by a variety of factors, including economic factors like income and expected income (11, 12), social factors such as the degree of social trust and social networks (13), psychological factors including self-perceived disease risk and self-perceived hazards (14, 15), and individual characteristics like age, gender, education level and self-assessed health (16–18). However, previous research overlooks the influence of health literacy on health investment intention.

Health literacy is the degree to which individuals have the ability to find, understand, and use information and services to inform health-related decisions and actions for themselves and others (19). High health literacy is empirically linked to a range of positive health-related behaviors and outcomes (20), such as improved health decision-making (21), proper medication use (22), decreased mortality and morbidity (23, 24), and enhanced communication with healthcare providers (25). Notably, the improvement of health literacy is a key driver in prompting individuals’ awareness and engagement with preventive healthcare services (24, 26). Conversely, low health literacy is often associated with an increased dependence on treatment and emergency services over preventive measures (27).

The relationship between health literacy and health investment intention remains somewhat controversial. Theoretically, health literacy endows individuals with essential skills to comprehend health information, thereby empowering them to accurately evaluate their health needs and improve the quality of health-related decisions, which can potentially increase their health investment intention. However, empirical evidence suggests a paradox: while individuals often acknowledge the benefits of preventive healthcare, they frequently show reluctance to incur its costs (28). This observation raises questions about the straightforward link between high health literacy and increased health investment intention. To clarify the relationship, further empirical research is necessary.

This study investigates the health literacy and health investment intention of employees in a petrochemical company through a cross-sectional analysis. It aims to examine the relationship between the employees’ health literacy and health investment intention, thereby providing some implications to enhance both the occupational health of petrochemical employees and the health human capital of the petrochemical industry.

## 2 Article types

The comprehensive cross-sectional analysis of 39,911 employees in a major petrochemical company in China addresses a relatively under-explored area - the impact of health literacy on health investment decisions in a high-risk occupational setting, and aligns profoundly with the objectives of ‘Public Health Education and Promotion’. We provide empirical evidence supporting the positive correlation between health literacy and health investment intention, highlighting the importance of enhancing health literacy as a means to encourage proactive health investment behaviors. The study’s exploration of the general yet modest health investment intentions among employees also shows the complexities of translating health literacy into actual health-promoting behaviors. This finding reveals health education should not only focus on popularizing health knowledge but also on facilitating its practical application and internalization into health beliefs and actions. Additionally, the recommendations provided in the manuscript for personalized health education, focusing on chronic diseases and employing behavioral economics principles like ‘nudge’ strategies, are innovative and align well with the latest trends in health education and promotion.

## 3 Materials and methods

### 3.1 Study design and participants

This study employed a cross-sectional study design. Data were collected from one of China’s largest petrochemical companies. The sole inclusion criteria was that the respondent must be an in-service employee of the petrochemical company. The primary objective of this study is to examine the relationship between employees’ health literacy and their intentions to invest in health. We will test the following research hypotheses: H_a_: Health literacy has a significant positive effect on health investment intentions. H_0_: Health literacy has no significant positive effect on health investment intentions. The secondary objective of this study is to investigate the current state of health literacy and health investment intentions among employees in a petrochemical company.

Prior to initiating the formal investigation, a preliminary survey was carried out among selected employees within a subsidiary, which aimed to provide a comprehensive evaluation of the questionnaire’s design. The survey respondents, comprising employees with diverse educational backgrounds, provided feedback that demonstrated a clear understanding of the questionnaire’s design and layout.

In the formal investigation phase, the survey questionnaire was deployed on the company’s internal website from April 1, 2022, and remained accessible for a period of 1 week. During this time, employees were afforded the opportunity to voluntarily complete the questionnaire at any time and from any location. The duration to complete the questionnaire was limited to 5 to 20 min. Out of an approximate total of 108,000 employees, 45,024 individuals voluntarily participated in the survey. To maintain the integrity of the responses and discourage dishonesty, respondents were limited to switching away from the questionnaire screen no more than twice during completion. Out of the 45,024 questionnaires returned, 5,113 were disqualified due to cheating, yielding an effective response rate of 88.64%. Consequently, the valid sample size for this study amounted to 39,911 respondents. This study adhered to the guidelines of the Declaration of Helsinki and received approval from the Ethics Committee of Traditional Chinese Medicine Hospital in Dongying District (No. ZYY-LL-006).

### 3.2 Measure

The questionnaire used in this study includes three sections.

1. Socio-demographic information: This section gathers data on respondents’ gender, age, income, marital status, education level, occupation, and presence of any chronic diseases. Table 1 shows the variable assignment table.
2. Health Investment Intention: It is measured using two questions. The first question is, “When you are in good health, are you willing to invest in your health? (Such as: pay for health care products, pay for health insurance, pay for health management costs)” with responses coded as a binary variable: “unwilling = 0, willing = 1.” As the construct of health investment intention is concrete, employing a single-item “global” measure is more concise, efficient and allows respondents to comprehensively consider all facets of the concept and their personal preferences (29). The second question is, “How much are you willing to pay annually to invest in your health” with responses coded as a multiple categorical variable: “0 yuan = 0, ≤2,000 yuan = 1, 2,000–5,000 yuan = 2, 5,000–10,000 yuan = 3, >10,000 yuan = 4.” For statistical analysis, it is treated as a continuous variable in the linear regression model.
3. Health Literacy: The study employs the “2020 National Health Literacy Monitoring Questionnaire,” developed by the National Health Commission of China, to measure health literacy. This questionnaire includes three aspects: Basic Knowledge and Attitudes, Health-Related Skills, and Healthy Behaviors and Lifestyles. Further, it can also be divided into six dimensions: Scientific Views of Health, Health Information, Infectious Disease, Chronic Disease, Safety and First Aid, and Primary Medical Care. The questionnaire’s maximum score is 73, with respondents answering over 80% of the questions correctly considered qualified. In the statistical analysis, the total scores were treated as a continuous variable in the linear regression model, while the qualification status was used as a binary variable in the logistic regression model.

**Table 1:** Variable assignment table.

Variables	Definition
Gender	Male = 1
	Female = 2
Age	<30 = 1
	30 ~ 39 = 2
	39 ~ 49 = 3
	≥50
Marriage Status	Married = 1
	Single = 2
	Divorced or Widowed = 3
Educational level	Junior high school = 1
	High school/Vocational high school = 2
	Bachelor’s degree/junior college = 3
	Master or higher = 4
Occupation	Skilled Operator = 1
	Technicians = 2
	Manager = 3
Income	<2000 = 1
	2000 ~ 4,000 = 2
	4,000 ~ 6,000 = 3
	≥6,000 = 4
Chronic Diseases	Have Chronic Diseases = 1
	No Chronic Diseases = 2

### 3.3 Statistical analysis

This study utilized several statistical analysis methods, including descriptive analysis, Chi-square test, logistic regression, and multiple linear regression. The Chi-square test is applied to identify differences in health investment intentions among different groups. Logistic regression is employed to assess the effect of health literacy on health investment intention. Additionally, multiple linear regression is used to confirm the consistency and robustness of this relationship. All statistical analyses are performed using SPSS 22.0 (30). Results will be considered statistically significant if rejecting Alpha at *p* < 0.05.

## 4 Results

### 4.1 Socio-demographic characteristics

The socio-demographic characteristics of the respondents are shown in Table 2. The majority of the respondents (74.8%) fell within the young to middle-aged category, aged 30–49. Over half of the employees (55.4%) held a bachelor’s degree or higher. Skilled operators constituted the majority (60.2%) of the respondents. Notably, the average monthly household disposable income for the vast majority (91.0%) was below 6,000 yuan. Most of the employees (72.1%) had at least one chronic disease.

**Table 2:** Basic information of the petrochemical employees.

Categories	Frequency	Rate (%)
Gender		
Male	23,436	58.72
Female	16,475	41.28
Age		
<30	711	1.78
30 ~ 39	7,763	19.45
39 ~ 49	22,072	55.30
≥50	9,365	23.47
Marriage Status		
Married	36,232	90.78
Single	1,077	2.70
Divorced or Widowed	2,602	6.52
Educational level		
Junior high school	647	1.62
High school/Vocational high school	17,141	42.95
Bachelor’s degree/junior college	20,597	51.61
Master or higher	1,526	3.82
Occupation		
Skilled Operator	24,037	60.23
Technicians	9,341	23.40
Manager	6,533	16.37
Income		
<2000	1,238	3.10
2000 ~ 4,000	18,244	45.71
4,000 ~ 6,000	16,821	42.15
≥6,000	3,698	9.04
Chronic Diseases		
Have Chronic Diseases	28,788	72.13
No Chronic Diseases	11,123	27.87

### 4.2 Health literacy

Among the surveyed employees, 52.1% (20,782 individuals) met the qualification criteria, with an average score of 56.11. The scores and qualification rate across three aspects and six dimensions of health literacy are shown in Table 3. Notably, the Chronic Disease dimension exhibited the lowest qualification rate at 33.0%.

**Table 3:** Qualified rate and scores of health literacy.

Categories	Scores (x̄ ± s)	Qualified rate (%)
Health literacy	56.11 ± 10.34	52.1
Three Aspects		
Basic Knowledge and Attitudes	25.29 ± 4.42	60.9
Healthy Behaviors and Lifestyles	19.10 ± 4.15	65.9
Health-Related Skills	11.71 ± 2.91	46.3
Six Dimensions		
Scientific Views of Health	9.09 ± 1.90	70.3
Health Information	5.76 ± 1.63	64.8
Infectious Disease	6.15 ± 1.74	68.7
Chronic Disease	11.05 ± 2.58	33.0
Safety and First Aid	12.47 ± 2.60	86.0
Primary Medical Care	11.59 ± 2.56	41.2

### 4.3 Health investment intention

In terms of health investment intention, the results indicated a majority of employees (71.5%) intended to invest in their health. Nonetheless, a notable proportion (34.5%) preferred the minimum investment option, under 2,000 RMB, as detailed in Table 4.

**Table 4:** Amount and rate of health investment intention.

Categories	Amount	Rate (%)
No Investment Intention	11,392	28.5%
Less than RMB 2,000	13,818	34.7%
From 2,000 to 5,000 RMB	10,868	27.2%
From 5,000 to 10,000 RMB	3,006	7.5%
Over 10,000 RMB	827	2.1%

### 4.4 Differences of health literacy and health investment intentions in socio-demographic variables

After the chi-square test, it was found that there is a significant correlation between the qualified condition of health literacy, the health investment intentions, and socio-demographic variables such as gender, age, marital status, educational level, occupational type, income, and the presence of chronic diseases (*p* < 0.05), as detailed in Table 5.

**Table 5:** Socio-demographic differences in health literacy and health investment intention.

Variables	Categories	Qualified in health literacy	Have health investment intentions
Gender	Male	11,051 (47.2%)	15,507 (66.2%)
	Female	9,731 (59.1%)	13,012 (79.0%)
	ꭓ2	549.971**	778.678**
Age	<30	376 (52.88%)	487 (68.5%)
	30 ~ 39	3,831 (49.35%)	5,475 (70.5%)
	40 ~ 49	12,057 (54.63%)	16,234 (73.6%)
	≥50	4,518 (48.24%)	6,323 (67.5%)
	ꭓ2	135.923**	125.030**
Marital status	Married	19,051 (52.6%)	26,054 (71.9%)
	Unmarried	5,179 (48.0%)	699 (64.9%)
	Divorced or Widowed	1,214 (46.7%)	1,766 (67.9%)
	ꭓ2	41.475**	42.718**
Educational level	Junior High School	184 (28.44%)	360 (55.6%)
	High School	7,571 (444.17%)	11,345 (66.2%)
	Bachelor	11,978 (58.15%)	15,588 (75.7%)
	Master or higher	1,049 (68.74%)	1,226 (80.3%)
	*ꭓ*2	1048.972**	552.028**
Occupation type	Skilled Operators	11,179 (46.51%)	16,367 (68.1%)
	Technicians	5,950 (63.70%)	7,127 (76.3%)
	Managers	3,635 (55.92%)	5,025 (76.9%)
	ꭓ2	842.773**	336.360**
Income	<2000	554 (44.75%)	617 (49.8%)
	2000 ~ 4,000	9,156 (50.19%)	12,647 (69.3%)
	4,000 ~ 6,000	9,047 (53.78%)	12,382 (73.6%)
	≥6,000	2,025 (56.13%)	2,873 (79.6%)
	ꭓ2	96.094**	480.836**
Chronic Diseases	have Chronic Diseases	14,798 (51.2%)	20,254 (70.1%)
	no Chronic Diseases	5,984 (54.4%)	8,265 (75.1%)
	ꭓ2	32.601**	99.736**

Statistical significance was set at *p < 0.05; **p < 0.01.

### 4.5 Effect of health literacy on health investment intention

A binary logistic regression was conducted with health investment intention as the dependent variable and health literacy qualified condition as the independent variable, controlling socio-demographic factors such as gender, age, marital status, education, occupation, income, and chronic diseases. The results, shown in Table 6, indicate that all these factors significantly influence health investment intentions. Notably, respondents with qualified health literacy exhibited a higher health investment intention (*OR* = 1.474, *p*<0.05) compared to those with unqualified health literacy.

**Table 6:** Logistic regression model for influencing factors of health investment intention.

Variables	*B*	*S.E.*	*p*	*OR*
Gender (ref: female)	−0.638	0.026	0.000**	0.528
Age. (ref:<30)				
30–39	0.055	0.098	0.571	1.057
40–49	0.296	0.099	0.003*	1.344
≥50	0.227	1.01	0.024*	1.255
Education level (ref: junior high school)				
High school	0.213	0.083	0.010**	1.237
Bachelor	0.568	0.085	0.000**	1.766
Master or higher	0.838	0.111	0.000**	2.311
Marital status (ref: married)				
Unmarried	−0.270	0.077	0.000**	0.764
Divorced or widowed	−0.169	0.045	0.000**	0.844
Occupation type (ref: skilled operators)				
Technicians	0.066	0.033	0.049*	1.068
Managers	0.223	0.036	0.000**	1.249
Income (ref: <2000)				
2000 ~ 4,000	0.701	0.061	0.000**	2.016
4,000 ~ 6,000	0.965	0.061	0.000**	2.624
>6,000	1.269	0.072	0.000**	3.557
Chronic Disease (ref: no chronic diseases)	−0.079	0.027	0.004*	0.924
Health literacy (ref: not qualified)	0.388	0.023	0.000**	1.474

Statistical significance was set at *p < 0.05; **p < 0.01.

To further examine the robustness of the relationship between health literacy and health investment intention, the study employed alternative measures for both the independent and dependent variables and developed a multiple linear regression model. In this approach, health investment intention was quantified as a continuous variable, scaled from 0 to 4, to reflect the intensity of intention, and was used as the dependent variable. Health literacy score, instead of health literacy qualified condition, was used as the independent variable. The findings reveal that health literacy had a significantly positive effect (*β* = 0.086, *p*<0.05) on health investment intention. (Table 7).

**Table 7:** Multiple regression model for the effect of health literacy on health investment intention.

Variables		The degree of health investment intention
Control variables		
Independent Variable	Health Literacy Score	0.086**
*R^2^*		0.083

Statistical significance was set at *p < 0.05; **p < 0.01.

## 5 Discussion

It is crucial to improve occupational health among employees in the petrochemical industry. Previous studies have demonstrated health literacy and health investment intention significantly impact their health status. This study focuses on assessing the health literacy and health investment intentions of employees in a petrochemical company, aiming to clarify their relationship. The study reveals three key findings, which are outlined as follows.

First, the study showed that the qualified rate of health literacy among employees from the petrochemical company was 52.1%, surpassing the average level (23.15%) of Chinese citizens in 2021 (31). This discrepancy can be attributed to two reasons. On one hand, more than half of the employees (55.4%) in our study held a bachelor’s degree or above. Previous studies have confirmed higher educational levels often correlate with increased health literacy (32, 33). On the other hand, the company had implemented a long-term health education and promotion program before our investigation, which may play a crucial role in enhancing health literacy (34). Among different aspects and dimensions of health literacy, “Chronic Disease” (33.0%) demonstrated the lowest qualified rates, which might contribute significantly to the high incidence (72.1%) of chronic diseases among the employees since previous studies have indicated that limited health literacy regarding specific diseases can substantially increase their prevalence (24, 35).

Second, the results showed that 71.5% of the respondents had the intention to invest in their health, but the intended investment amounts were not high. Specifically, almost half of those who had health investment intention opted for the minimal investment bracket, under 2,000 RMB annually. The limited ability and motivation are two possible reasons for the limited amount of health investment. As for ability, low disposable income may be a constraint on higher health investment, since prior studies found those with low income frequently encounter financial hurdles in pursuing activities promoting health (36), such as maintaining a balanced diet, engaging in regular exercise, and avoiding high-risk activities (37, 38). In this study, 91% of respondents reported a household *per capita* disposable income below 6,000 RMB monthly. This figure is notably lower than the average monthly wage of 7,897 yuan reported in the Shandong Statistical Yearbook, which significantly restricts their ability to increase health investments ((39), p.7, Tables 1, 2). As for motivation, the intrinsic characteristics of health as a form of capital may diminish investment motivation. First, healthy human capital is acquired inborn, declines slowly, and diminishes imperceptibly, making individuals undervalue the potential disease susceptibility and seriousness (40, 41). Second, the return on investment of health human capital always has a certain delay, which will exacerbate the myopia effect and lead individuals to favor current utilities and undervalue perceived long-term health benefits (41). Additionally, the company offers both basic medical social insurance and supplementary medical insurance to all employees. Given the cost sharing mechanism between employer and employee in China’s social medical insurance, this provision may diminish the respondents’ intention to purchase commercial medical insurance, leading to a generally low level of health investment intention.

Third, a final but important finding of this study is that health literacy does positively influence health investment intention, though the strength of this effect is limited. The conversion of the standardized regression coefficient (*β*) to an effect size *d* of 0.2746, and the conversion of the Odds Ratio (*OR*) to an effect size *d* of 0.2139, both indicates that health literacy exerts a small effect (ranging from 0.2 to 0.4) on health investment intentions (42, 43). In our view, this observed limited effect may stem from two aspects. On one hand, health literacy may act as a distal variable, indirectly influencing health investment intention through other mediating variables (44). For example, health beliefs, being closer in the causal chain, might have a more direct impact on health investment intention and could mediate the effect of health literacy (45). On the other hand, the scale employed in this study focuses on evaluating employees’ specific health knowledge, thereby may not comprehensively capture the connotation of health literacy, though it is widely used in China. However, health literacy is a multifaceted concept that encompasses the ability to apply health information and critical thinking skills for informed health decisions (46). These facets, potentially more influential in shaping health investment intention, were not adequately represented in this study.

## 6 Limitations

While this study yielded noteworthy findings, it is not without limitations. Firstly, the non-random sampling raises concerns about the generalizability of the conclusions. This study is limited by its single-institutional scope and cultural context, which constrain its generalizability to other occupational settings and population groups within the country. All participants were from a single petrochemical company that had implemented a health education and promotion program before the investigation, probably resulting in higher-than-average levels of health literacy among those surveyed. Furthermore, the company provides employees with both basic medical social insurance and supplementary medical insurance, which may contribute to a low level of health investment intention. The next phase of research should focus on addressing the limitation of inadequate sample representation by conducting comprehensive studies across entire populations and multiple countries. Secondly, potential selection bias may exist due to the response rate. More than half of the employees declined to participate, potentially introducing systematic bias into the conclusion. Thirdly, as previously mentioned, the health literacy measurement scale predominantly focused on assessing health knowledge, neglecting the assessment of the ability to apply health information and make informed health decisions, which may decrease the validation of measurement. Moreover, there are inherent measurement errors associated with using a single item to measure the health investment intention. It is advisable to develop multi-level measurement tools that can more accurately define health investment intention and its specific components in the future research. Finally, given the cross-sectional design of the study, it is unable to establish causality between health literacy and health investment intention. Additionally, the omission of certain confounding variables could introduce endogeneity issues into the analysis.

## 7 Conclusions and implications

This study undertook a cross-sectional survey involving 39,911 employees from a petrochemical company to evaluate their health literacy and health investment intention, as well as to clarify the relationship between them. The results revealed that the employees’ health literacy significantly exceeds the national average for Chinese citizens, yet the qualified rate of Chronic Disease dimension is relatively low. A majority of employees have the intention to invest in health, albeit with a modest amount. Furthermore, the study demonstrates that while health literacy does positively influence health investment intention, this effect is somewhat limited.

Based on the aforementioned conclusions, the study proposes several recommendations. First, health education and promotion should focus on improving the employees’ health knowledge regarding chronic disease. Personalized health education, informed by the outcomes of employees’ physical examinations, should be implemented, emphasizing specific aspects like prevention, early detection, management, and treatment of chronic diseases. Moreover, these programs should incorporate interactive training methods, such as group discussions and question-and-answer sessions, to increase employee engagement and learning efficacy.

Second, health education should focus not only on spreading health knowledge but also on facilitating its internalization into health beliefs. Employing ‘nudge’ strategies from behavioral economics could help overcome psychological barriers. For example, according to the principle of loss aversion, where individuals generally prefer avoiding losses over acquiring similar gains, health education could be more effective in underscoring the negative consequences (such as illness, increased medical costs, and reduced quality of life) of unhealthy behaviors through concrete examples, rather than merely focusing on the benefits of healthy practices.

Finally, it is imperative for both the company and government to enhance the financial capacity of employees, as income is a pivotal determinant of their ability to invest in health, either through direct or indirect means. The company could establish a dedicated health investment fund aimed at offsetting the costs of preventive healthcare for employees. This fund would cover expenses related to regular health check-ups, vaccinations, and wellness programs. Concurrently, governments could offer incentives to encourage healthy behaviors in the workforce, such as tax benefits for participating in health programs, financial aid for obtaining health insurance, and subsidies for health-promoting activities. Implementing these strategies would not only improve the health outcomes of employees but also potentially reduce long-term healthcare costs.

## Data availability statement

The raw data supporting the conclusions of this article will be made available by the authors upon reasonable request and with permission of the data owners.

## Ethics statement

This study adhered to the guidelines of the Declaration of Helsinki and received approval from the Ethics Committee of Traditional Chinese Medicine Hospital in Dongying District (No. ZYY-LL-006). The participants provided their written informed consent to participate in this study.

## Author contributions

DW: Writing – review & editing, Writing – original draft. YW: Writing – review & editing, Investigation, Data curation. HM: Writing – review & editing, Validation, Supervision. SZ: Writing – review & editing, Writing – original draft, Methodology, Investigation, Funding acquisition, Data curation.
